# Developmental enamel defects and their relationship with caries in adolescents aged 18 years

**DOI:** 10.1038/s41598-023-31717-2

**Published:** 2023-03-27

**Authors:** Dorota Olczak-Kowalczyk, Norbert Krämer, Dariusz Gozdowski, Anna Turska-Szybka

**Affiliations:** 1 Department of Paediatric Dentistry, Medical University of Warsaw, Binieckiego 6 St, 02-097 Warsaw, Poland; 2 Department of Pediatric Dentistry, Medical Center for Dentistry, University Medical Center Giessen and Marburg, Campus Giessen, Schlangenzahl 14, 35392 Giessen, Germany; 3 Department of Experimental Statistics and Bioinformatics, Warsaw University of Life Science, Nowoursynowska 166, 02-787 Warsaw, Poland

**Keywords:** Dental diseases, Diseases

## Abstract

Randomly selected 1,611 individuals aged 18 years formed a nationally representative sample from all provinces of Poland. Developmental defects of the enamel (DDE) and caries were assessed using the modified DDE index, molar incisor hypomineralisation (MIH) Treatment Need Index (MIH-TNI), FDI and WHO criteria by 22 trained and calibrated dentists. T-test was used for comparing group means. A simple and multiple logistic regression tests were used to assess the relationship between DDE and caries severity expressed as DMFT (p < 0.05). The prevalence of DDE was 13.7%. Demarcated opacities (DEO) were the most frequent (9.65%); 4% had diffuse opacities (DIO) and 1.5% had hypoplasia. MIH was diagnosed in 0.6% patients. The prevalence of caries was 93.2%, with mean DMFT of 6.50 ± 4.22. The DMFT value was 7.52 ± 4.77 in the group of patients with demarcated opacities (DEO); 7.85 ± 4.74 with diffuse opacities (DIO) and 7.56 ± 4.57 with enamel hypoplasia, respectively. There was a significant relationship between caries severity and DDE (p < 0.001), DEO (p = 0.001) and DIO (p = 0.038), and between DDE and DMFT index (p < 0.001). The results obtained in the study proved the significant relationship between DDE and DMFT in 18-year-olds, the assessment of which was the aim of the study.

## Introduction

Enamel morphogenesis is a complex process which starts with enamel matrix protein secretion, followed by mineralisation and maturation. The process begins at the cusps and incisal parts of the crowns, progressing towards the cervical areas of the teeth. Disturbances in different stages of enamel formation may result in a range of macroscopic and structural changes^1^. Defective formation of the enamel matrix leads to hypoplasia, a quantitative defect which is clinically manifested by generalised enamel thinning or pitting defects, grooves or local loss of enamel. Defective calcification leads to hypomineralisation, a qualitative enamel density defect presenting in vivo as changes in colour and translucency of the enamel in the form of either demarcated opacities, with clearly defined margins, or diffuse opacities, without clear borders^1^. Developmental Defects of Enamel (DDE) are clinically manifested as white/cream enamel opacity (demarcated opacities (DEO), diffuse opacities (DIO), hypoplasia (Hypo)), associated with opacity or their combination^1–3^.

The possible aetiology of DDE includes a number of genetic, systemic, environmental and local factors^1–4^. Fluoride level in drinking water in Poland is below 0.5 ppm F/l. The risk of fluorosis also seems to be low^5^. Studies demonstrated a relationship between history of certain systemic diseases in early childhood (anemia, rubella, rickets, tetany, kidney and liver diseases, allergy, diarrhoeas, as well as administration of tetracycline and iron supplements), and an increased incidence of DDE in permanent dentition^1^. The influence of local and sociodemographic factors is also suggested^4^. Epidemiological data shows high rates and severity of caries in the country. Over 16 years (2001 vs. 2017), the prevalence of caries among 18-year-olds decreased by only 4.2% (from 97.4% to 93.2%), and the mean DMFT decreased by 0.8 (from 7.3 to 6.5)^6^. Dental caries is a biofilm-mediated, sugar-driven, multifactorial, dynamic disease that results in the phasic demineralization and remineralization of dental hard tissues^7^. The development of the disease is modified by many factors, including socio-demographics and eating habits, oral hygiene practices and fluoride delivery to the oral environment.

A number of studies point to the possible relationship between DDE and the higher caries experience caries^8–12^. Developmental enamel defects may play an important role in increasing the susceptibility to caries development^13–17^. Increased rates of caries in teeth presenting with opacity and hypoplasia are due to increased microporosity and poor mineralisation, as well as increased accumulation of dental plaque^8,18,19^.

Psychological well-being and Oral Health Related Quality of Life (OHRQoL), may be associated with aesthetic perception of teeth affected by the abnormal discoloration and tooth morphology associated with developmental enamel defects^17,19,20^. Some adolescents of Sujak et al.^20^ study were dissatisfied with the condition of teeth affected by DDE. Among subjects who expressed dissatisfaction, 18.8% reported covering their mouths when smiling, 8.7% avoided going out with friends and 39.1% had consulted their dentists^20^. Results of studies indicated that the presence of DDE may cause negative impacts on one’s perception of oral health and on their daily performance^21,22^. The patients can suffer pain, difficulties in eating, tooth brushing, or in anaesthetising. Dental fear and anxiety, as well as dental behaviour problems as a consequence of DDE, have been reported^22^.

There is limited information about DDE in adolescents^18,20,23,24^. The majority of studies concerning enamel defects have investigated children and young adolescents up to 12–14 years of age only^8–11,15–17,21^. Therefore, very little information is available about the extent and severity of DDE in full permanent dentition taking into account longitudinal changes with time in the epidemiological profile of DDE. This indicates that older age groups should be investigated as well. Comparing to healthy individuals, adolescents affected by DDE would need intensified professional prevention and treatment.

The aim of the cross-sectional study was to assess the potential relationship between different types of developmental defects of the enamel (DDE) and DMFT in a total of 1611 adolescents aged 18 years. The outcome variable was DDE and caries severity.

## Methods

This was a cross-sectional study involving questionnaire and clinical examinations. Adolescents aged 18 years (both genders) were selected based on three-layered randomisation to form a representative sample of population, and enrolled in an epidemiological cross-sectional study conducted from October to November 2017 as part of monitoring of the oral health status of the Polish population^4,6^.

### Sample size

In all sixteen provinces of Poland, administrative divisions of second level (counties) and third level (communes that are classified as urban or rural) were randomly chosen. The population study group was defined by a three-stage cluster sampling procedure: selection of states (the first large cluster), then selection of samples of schools (second-level cluster), followed by samples of groups (third-level cluster) and finally samples of 18-year-old adolescents. A minimum sample size as 1210 subjects was calculated based on the total number of 18-year-old adolescents living in Poland and their developmental enamel defects prevalence (15% ± 2% margin of error at 95% confidence level).

**Figure 1:**
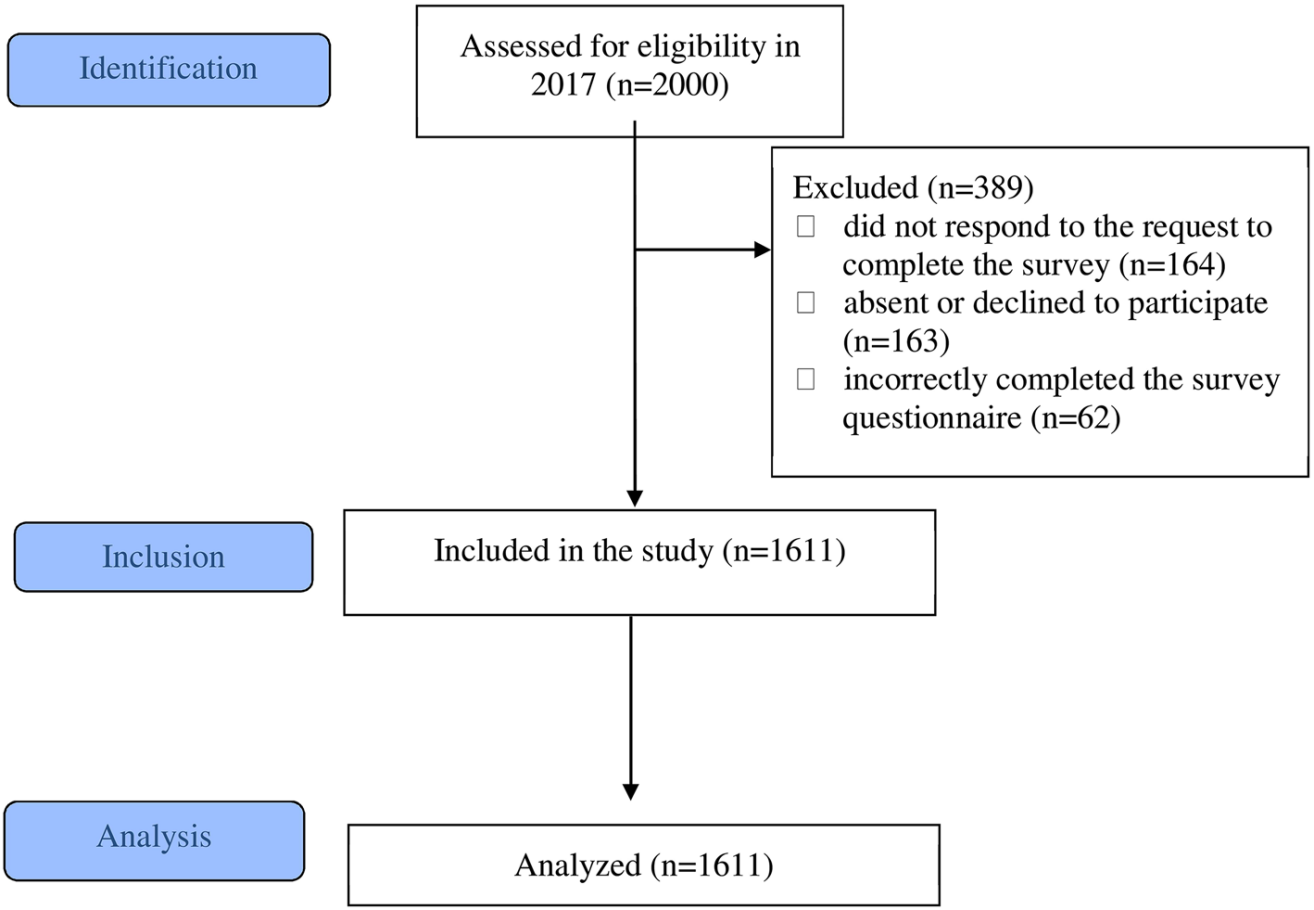
Study enrolment flow chart.

A total of 2000 adolescents attending twenty-five schools were originally invited to participate in the study. Ultimately, there were 1611 participants from twenty-three schools. Figure 1 shows study enrolment. 8% of 18-year-olds failed to fill in the questionnaire, 3% filled it incorrectly and 8% were absent during dental examination or refused to be examined.

The attendance in the survey was voluntary; however, the approval from the concerned school authorities was obtained. The inclusion criteria were: age, written consent for participation in the study and a completed questionnaire containing questions about socioeconomic factors (place of residence, level of parental education, economic status), as well as the presence at school on the day of the examination. Adolescents with disability and illnesses were excluded from the study. Immigrants were excluded from the population sample because they might have different histories and demographics. Adolescents who were currently using or had previously used permanent orthodontic appliances were excluded as well.

The survey was conducted with the use of a questionnaire including questions about gender, place of residence (urban/rural), parents’ education level, family economic status.

The Strengthening the Reporting of Observational Studies in Epidemiology (STROBE) guidelines for reporting observational studies was used and followed^25^.

### Clinical examination

Clinical examination of teeth was performed in artificial light ((Dental 3W Mobile Portable Surgical Exam Light Medical Examination Lamp JSF-JC02) using plane mouth mirrors (Hinte; ISO 9001:2008, CE MARK) and CPI (Community Periodontal Index) probe (Hinte; CE and ISO 9001:2015) in accordance with the WHO standard for epidemiological surveys (0.5 mm ball tip)^26^. Prior to the clinical examination the participants brushed their teeth. Caries was assessed using WHO criteria for the permanent dentition (DMFT)^26^. Developmental enamel defects involving the labial/buccal surface of the teeth, based on the appearance and extension were assessed according to the modified Developmental Defects of Enamel (mDDE) index^27^. DDE was classified as demarcated white, yellow or brown opacities, diffuse opacities, and hypoplasia. Enamel opacity was considered to be a distinct change in enamel translucency. A diagnosis of enamel hypoplasia was made when there was evidence of deficiency in enamel formation seen clinically as localized or generalized pits and grooves on the surfaces of teeth. Demarcated enamel opacities, surface breakdowns of hypomineralised enamel and atypical fillings and tooth extractions due to described by Weerheijm et al.^28^ in 2001 molar-incisor hypomineralisation (MIH) were diagnosed according to the new 4-step MIH Treatment Need Index (MIH-TNI)^29–31^. In cases of stainless steel crowns (SSCs) on molars and/or fillings/composite reconstructions in anterior teeth the patient was consulted as to the cause of such a reconstruction—trauma, caries, enamel defect—and appropriate annotation was made on the patient’s chart. Traumatized anterior teeth and DDE/MIH-associated defects were not scored in the DMFT index. Hypomineralised lesions with a diameter < 1 mm were not recorded^32^. Other enamel defects were excluded.

Dental evaluation was performed by 22 trained and calibrated dentists. Before the study, theoretical and clinical training and calibration focused on carious lesions, DDE and MIH was undertaken with all 22 dentist and supervised by an experienced dentist (DO-K).

The theoretical training provided information about study design, indices and diagnostic principles and was followed by a clinical course in which each of the investigators of sixteen teams from each province of Poland, consisting of two dentists, specialists in paediatric dentistry with many years of experience, examined 18-year-olds under supervision of an experienced dentist (DO-K). Each paediatric dentist (examiner) independently examined the same group of ten patients. To determine the reliability of all investigators inter- and intra-examiner Kappa values were calculated; they were found to be in a good to excellent order of magnitude. Approximately 5% of the children were randomly selected for re-examination to continuously monitor the intra- and interexaminer reliabilities during the surveys. Mean inter-rater reliability between the reference examiner and other examiners was 0.898 (range from 0.857 to 1.000) for carious lesions, 0.949 (range from 0.872 to 1.000) for DDE, whereas mean intra-examiners reliability was 0.988 (range from 0.963 to 0.999) and 0.986 (range from 0.952 to 1.000), respectively. The majority of the participants had access to municipal drinking water with a low level of fluoride (< 0.5 mg/ml)^5^. Furthermore, drinking water was sampled in all regions to assess fluoride content. An ion-selective electrode (Orion 9609) was used to determine water content of fluoride in the residential areas of participants by adding standard NaF solutions (fluoride standard 100 ppm F by Orion) twice. The detection rate of this method is 0.02 mg/l. The relative standard deviation did not exceed 7%. The average reading was calculated. The adolescents were asked about aesthetic and treatment problems caused by their enamel developmental defects.

### Statistical analysis

Statistical analysis was performed using Statistica 12.0 (StatSoft) software (p < 0.05). T-test was used for comparing group means. The descriptive statistical analysis of the DDE and caries severity data included the determination of prevalence rates according to cut-off (DMFT = 0; DDE = 0). Adolescents with at least one DDE were categorised as group DDE ≥ 1; otherwise, subjects with no DDE were scored as free of DDE. The components of the caries appearance and DDE were determined separately, and mean values (standard deviation) were calculated. Relationships between pairs of selected variables were evaluated using Spearman's rank correlation coefficient. A simple and multiple logistic regression tests were used to assess the relationship between DDE, which was treated as independent variables, and dental caries which were treated as binomial dependent variable. The results were presented in the form of odds ratios (OR—odds ratio) for simple logistic regression, and adjusted odds ratios (AOR) for multiple logistic regression as well confidence intervals (CI at 95% confidence level) for OR. Socioeconomic factors were considered as confounders in AOR calculation. Significance level for all the analyses was set at 0.05.

### Ethical approval and informed consent

The study was approved by the Bioethics Committee of the Medical University of Warsaw (KB/134/2017). The study has been conducted in accordance with the World Medical Association Declaration of Helsinki (version, 2008). Informed consent was signed and obtained from each patient participated in the study.

## Results

A total of 1611 adolescents aged 18 years participated in the study. Socioeconomic data are presented in Table 1.

**Table 1:** Characteristics of participants.

Parameters		Total N / % (95% confidence interval)	Patients with DDE and caries	Patients with DDE only (without caries)
Socio-demographic factors				
Gender	Female	847/52.6% (50.1–55.0)	96/11.3% (9.3–13.7)	6/0.7% (0.2–1.5)
	Male	764/47.4% (45.0–49.9)	110/14.4% (12.0–17.1)	8/1.0% (0.5–2.1)
	p		0.130	0.511
Place of residence	Urban area	797/49.5% (47.0–51.9)	110/13.8% (11.5–16.4)	9/1.1% (0.5–2.1)
	Rural area	814/50.5% (48.1–53.0)	96/11.8% (9.7–14.2)	5/0.6% (0.2–1.4)
	p		0.230	0.274
Mother’s education	Basic/primary	47/3.3%	8/17.0% (7.7–30.8)	1/2.1% (0.0–11.3)
	Vocational	418/29.0%	54/12.9% (9.9–16.5)	2/0.5% (0.0–1.7)
	Middle	532/36.9%	76/14.3% (11.4–17.6)	4/0.8% (0.2–1.9)
	Higher	446/30.9%	49/11.0% (8.2–14.3)	5/1.1% (0.3–2.6)
	p		0.383	0.555
Socioeconomic status	Low	47/2.9%	4/8.5%(2.4–20.4)	0/0% (0.0–2.2)
	Average	896/55.4%	111/12.4% (10.3–14.7)	5/0.6% (0.2–1.3)
	High	382/23.7%	48/12.6% (9.4–16.3)	3/0.8% (0.2–2.3)
	p		0.720	0.769

*chi-square test; p < 0.05.

The prevalence of DDE was 13.7% (220 patients), with an average of 0.85 ± 3.34 teeth affected (Table 2). The most common type of DDE was demarcated opacities (155/1611; 9.65%) and diffuse opacities (65/1611; 4.0%), followed by enamel hypoplasia (25/1611; 1.5%). MIH was diagnosed in 0.6% patients (10/1611).

**Table 2:** The prevalence of DDE in 18-year-old adolescents.

Parameters	Participant’s n/N (%) (95% CI)	Teeth mean ± SD
m-DDE Index > 0	220/1611 (13.7%) (12.0–15.4)	0.85 ± 3.34
Demarcated opacity	155/220 (70.4%) (64.0–76.4)	0.50 ± 2.32
Diffuse opacity	65/220 (29.5%) (23.6–36.1)	0.32 ± 2.31
Enamel hypoplasia	25/220 (11.4%) (7.5–16.3)	0.03 ± 0.26
MIH	10/220 (4.5%) (2.2–8.2)	0.02 ± 0.32

*n* patients with DDE, *N* -total study group, *CI* confidence interval.

Prevalence of different types of DDE were significantly different (p < 0.05). However, mean number of teeth affected by these types of DDE was very low and the differences between various types of DDE were not significant (p > 0.05).

Table 3 shows comparisons of patients with DDE and caries vs. DDE only.

**Table 3:** Comparisons of patients with DDE and DMFT and its components vs. DDE only.

Parameters	Patients with DDE and caries n/N (%) (95% CI)	Patients with DDE Only n/N (%) (95% CI)	p (for comparisons patients with DDE and caries vs. DDE only
m-DDE Index > 0	206/1611 (12.8%) (11.2–14.5)	14/1611 (0.9%) (0.5–1.5)	< 0.001*
Demarcated opacity	144/1611 (8.9%) (7.6–10.4)	11/1611 (0.7%) (0.3–1.2)	< 0.001*
Diffuse opacity	57/1611 (3.5%) (2.7–4.6)	3/1611 (0.2%) (0.0–0.5)	< 0.001*
Enamel hypoplasia	23/1611 (1.4%) (0.9–2.1)	1/1611 (0.1%) (0.0–0.4)	< 0.001*
MIH	1/1611 (0.1%) (0.0–0.4)	0/1611 (0.0%) (0.0–0.2)	0.317

*Chi-square test; p < 0.05.

The studied sample of adolescents was not exposed to confounding factors such as fluorine content in the tap water. The fluoride level in the tap water was below 0.5 ppm at the time when the participants of the present study were born and living before 6-year-old^6^. Fluorine levels in drinking water sampled in the residential areas of participants measured by an ion-selective electrode (Orion 9609) averaged 0.24 ± 0.22 ppm (mgF).

The prevalence of caries was 93.2% (1,501 patients), with 6.50 ± 4.22 teeth affected (i.e. 23.4% of teeth present in the oral cavity). The components of the DMFT index as related to DDE are shown in the Table 4. Stainless-steel crowns were not placed for molars restorations.

**Table 4:** Mean values of DMFT scores and their components as related to DDE.

Parameters	Developmental enamel defects		p based on Mann–Whitney *U* test
	Present	Absent	
Mean ± SD			
DMFT	7.66 ± 4.68	6.35 ± 4.12	< 0.001*
DT	2.58 ± 2.88	1.99 ± 2.78	< 0.001*
MT	0.13 ± 0.46	0.14 ± 0.52	0.943
FT	4.95 ± 3.79	4.22 ± 3.44	0.011*

*DMFT* decayed, missing, filled primary teeth, *DT* decayed tooth, *MT* missing tooth, *FT* filled tooth, *SD* standard deviation.

*Mann–Whitney *U* test; p < 0.05.

As many as 56.9% of respondents were dissatisfied with their teeth, and every tenth respondent admitted that they avoided a smile due to teeth appearance. Dental diseases in 7.0% of participants were the cause of problems with eating hard food, and in 3.2% with chewing.

Logistic regression analysis did not confirm the relationship of DDE with caries prevalence (Table 5). When DMFT > 0, there was no significant relationship with DDE. However, significant differences were found between the means of DMFT (between DDE vs. without DDE; with demarcated vs. control; diffuse opacities vs. control).

**Table 5:** A relationship between DDE and DMFT (and components).

	DMFT > 0	DMFT
	N (%)	Mean ± SD
Total study group	1501/1611 (93.2%)	6.50 ± 4.22
Patients with DDE	206/220 (93.6%)	7.66 ± 4.68
Patients without DDE (control)	1255/1348 (93.1%)	6.35 ± 4.12
p (vs. control)		< 0.001*
OR (95% CI)	1.09 (0.61–1.95), p = 0.770	
AOR (95% CI)	1.14 (0.61–2.14), p = 0.672	
Demarcated opacities	155/168 (92.3%)	7.52 ± 4.77
p (vs. control)		0.001*
OR (95% CI)	0.88 (0.48–1.62), p = 0.688	
AOR	1.04 (0.53–2.06), p = 0.903	
Diffuse opacities	62/65 (95.4%)	7.85 ± 4.74
p (vs. control)		0.005*
OR (95% CI)	1.53 (0.47–4.97), p = 0.478	
AOR (95% CI)	1.20 (0.37–3.95), p = 0.759	
Enamel hypoplasia	24/25 (96.0%)	7.56 ± 4.57
p (vs. control)		0.155
OR (95% CI)	1.77 (0.24–13.29), p = 0.575	
AOR (95% CI)	1.49 (0.20–11.27), p = 0.698	
Patients with MIH	10/10 (100.0%)	7.10 ± 2.18
p (vs. control)		0.565
OR (95% CI)	1.56 (0.09–26.90) p = 0.758	
AOR (95% CI)	1.52 (0.07–22.31) p = 0.874	

p-values for the differences and OR and AOR vs. control (individuals without DDE).

*statistical significance p < 0.05.

Odds ratio of the chance of occurrence of dental caries in all types of DDE was not significantly different in comparison with control (i.e. individuals without DDE). However, the differences were significant between means of DMFT for patients with DDE vs. control; patients with demarcated opacities vs. control and for patients with diffuse opacities vs. control.

Spearman’s correlation did not confirm a relationship between socio-economic status, parents’ education or DDE.

Table 6 shows the Spearman correlation coefficients between DMFT and DDE (a division into various defects, their number or the presence or absence (0/1).

**Table 6:** Spearman’s correlation between DMFT and DDE.

	D (cavitated)	M (missing due to caries)	F (filled)	DMFT
Diffuse opacity (number of teeth)	0.051*	− 0.011	0.042	0.058*
Demarcated opacity (number of teeth)	0.082*	0.012	0.045	0.077*
Hypoplasia (number of teeth)	0.029	− 0.040	0.015	0.028
MIH (number of teeth)	0.028	− 0.001	− 0.005	0.022
Diffuse opacity (0/1)	0.052*	− 0.011	0.043	0.059*
Demarcated opacity (0/1)	0.080*	0.012	0.044	0.076*
Hypoplasia (0/1)	0.029	− 0.040	0.015	0.028
MIH (0/1)	0.028	− 0.001	− 0.005	0.022

*Statistical significance p < 0.05.

Due to the large sample size, although the correlations were weak, they turned out to be statistically significant. The number of teeth with DDE did not increase the correlation with DMFT as it was similarly strong.

## Discussion

This is, to our knowledge, the first study investigating the developmental enamel defects and their relationship with caries in a nationally representative sample of adolescents aged 18 years living in all sixteen provinces of Poland. The study was a part of the Ministry of Health national programme assessing oral health in children and adolescents. Polish studies on larger populations have not yet been conducted.

In the past, monitoring studies on the oral condition of Polish population aged 18 years did not consider developmental enamel defects, thus, they cannot be compared. Enamel developmental defects (non-fluoride-related) were examined only in 15-year-olds in 2018 as part of Monitoring of oral health status Polish population in 2016–2020^23^. Lesions such as opacities, hypoplasia or discolorations were present in 130/992 (13.1%) participants, without statistical significance related to the place of residence or gender. Enamel opacities were the most commonly observed defects (12.5% of the total population), less frequently hypoplasia—13/992 (1.3%), discoloration—4/992 (0.4%) and combinations of DDE—6/992 (0.6%). The mean number of teeth with developmental defects aged 15 years was 1.38 ± 5.78. The prevalence of DDE in 18-year-olds in the present study was similar (13.7%). The same observation concerned the incidence of lesions—mostly opacities, less frequently enamel hypoplasia. Retrospective determination of the aetiology of enamel defects is difficult. The presence of demarcated opacity and hypoplasia in the form of isolated, sporadically located lesions indicates local causes. Diffuse opacities are usually found on the teeth with simultaneous enamel secretion and maturation, pointing to environmental aetiology, and are related to systemic causes. Traumatic damage or periapical inflammatory lesions in the primary tooth, or its early extraction, may disrupt normal matrix deposition or enamel mineralisation and, consequently, lead to enamel defect (a demarcated spot and/or hypoplasia) in the permanent tooth. The distribution of DDE according to teeth in regards to DDE in the entire examined population of Polish adolescents aged 18 years was addressed of our previous paper^4^.

The data from previous monitoring studies allow one to draw conclusions on the consequences of dental caries as a possible relationship with DDE. The 18-year-old adolescents in the study were aged 3 years in 2002, when the prevalence of caries among Polish children at that age was 56.2%, with a mean of 2.9 teeth affected^33^. In the same period (2002), caries was found in 86.9% of 6-year-olds, with 5.9 primary teeth involved^34^.

A number of studies have shown a positive relationship between DDE and the severity of caries^8–18,35^. The results of our study are consistent with previous studies that suggested that DDE increased the risk of dental caries, since the influence of enamel defects in the development of caries was observed^13,35,36^. Fotedar et al*.*^13^ demonstrated a significant association between caries and enamel opacity among 12- and 15-year-olds from India. The relationship between DDE and the severity of dental caries was also confirmed in our study, which demonstrated significantly increased caries severity (expressed as DMFT) in patients with DDE (7.66 ± 4.68 vs. 6.35 ± 4.12, p < 0.001). However, the severity of caries was increased in individuals presenting with qualitative enamel defects (demarcated and diffuse opacity) rather than those with quantitative defects (hypoplasia). Nevertheless, no significantly increased risk of caries among Polish adolescents in the presence of developmental enamel defects was shown, which may be due to the high prevalence of caries and a several-year retention time of teeth in the oral cavity. Some of these subjects will have their original DDE obliterated by caries, restoration or extraction. Burns & Holland^37^ concluded, based on their meta-analysis of studies assessing this relationship among 8–19-year-olds, that the positive correlation between caries and enamel defects may be considered to be a potential predictor of caries due to similar risk factors.

In relation to enamel defects, all the types of defects can be associated with dental caries. Enamel hypoplasia is more susceptible to dental caries since thinner porous enamel presents irregular surfaces with pits or grooves^35^. This enables higher bacterial biofilm accumulation and acid solubility with the subsequent progression of carious lesions^8,14^. Demarcated opacities are significantly associated with dental caries in permanent incisors and molars^38^.

Diffuse opacities are less susceptible to dental caries^17^. Dini et al.^17^ demonstrated a twofold lower risk of dental caries in children with diffuse enamel opacities compared to children with no or demarcated opacities. The term ‘diffuse opacities’ is used interchangeably with dental fluorosis when it is caused by an excessive intake of fluoride. The fluoride level in drinking water was below 0.5 ppm at the time when the participants of the present study were born and during the first years of their life. The low prevalence of diffuse opacities among participants in this study might be due to consumption of low level of fluoride in water. Another explanation might be the effect of remineralization. Compared with demarcated opacity, it is easier for diffuse opacities to be remineralized.

It is possible that the longitudinal observation of DDE differ among populations with different prevalence of DDE. When analysis was performed for the different types of DDE, higher prevalence of DEO was found compared to DIO. Reduction of frequency of diffuse opacities has been shown, similarly to a study by Wong et al.^39^. Demarcated opacities have distinct boundaries separating them from normal enamel, which are thus more unlikely to disappear due to mechanical and chemical factors. A question should be asked whether these are demarcated defects that promote the development of caries or whether dental caries develops due to the presence of causative factors of this disease in individuals with dental caries in primary dentition.

A positive association between enamel defects and dental caries was identified in meta-analysis of Vargas-Ferreira et al.^8^. Patients demonstrating DDE had higher pooled odds of having dental caries experience (OR 2.21; 95% CI 1.3; 3.54). Teeth affected by DDE have high sensitivity due to wear and high porosity, leading to lower mechanical strength and development of enamel breakdown and carious lesions^8^.

It is beyond doubt that the risk of differential misdiagnosis due to the lack of precise data on different fluorine sources in childhood, is an important limitation of epidemiological studies assessing the incidence of enamel opacities classified as dental fluorosis. It is important to emphasize that DDE and carious lesions were distinguished, diagnosed and recorded based on locations and surface features, following diagnostic criteria and recommendations^26–31^. Training and calibration of all examiners in the present study resulted in a good intra- and inter-examiner reliability.

Half of adolescents worry about their teeth. These findings are not in accordance with Sujak et al.^20^ who suggested that very few subjects were concerned about the appearance of their teeth, or were not aware of their teeth being different. In the Vargas-Ferreira et al.^8^ study children with DDE did not indicate any decrease in self-perception. However, this condition was associated with an impact on the functional limitation domain.

Due to the appearance and function of teeth, enamel developmental defects may affect the emotions and interactions of adolescents. Moreover, the prospect of a complex and long-lasting therapy may have adverse consequences^40^. DDE affected individuals whose self-esteem was determined by outward appearance, and those who relied on the way they were perceived and accepted by others^41^.

The strength of this study was large number of 18-year-old adolescents. A population-based sample was used, contrary to a clinical convenience sample. The presence of controlling for confounding such as socioeconomic factors to find its influence on findings was taken into account and analysed, in contrast to some other studies. When considering the methodology of the present study, it should be mentioned that the recording of carious lesions and DDE followed the most recently published recommendations^28–31^. The trained group of dental examiners showed good intra- and inter-examiner reliability values and good capacity to identify DDE and to discern the different types of DDE. Furthermore, restorations subsequent to carious lesions were delineated from DDE-related restorations, i.e. atypical restorations due to DDE were not scored as caries-associated restorations and, hence, were not part of the F-component and the DMF index. Furthermore, this study was conducted following STROBE guidelines for reporting observational studies^25^.

It is important to mention that the cross-sectional observational epidemiological design of the study does not form a basis to establish the temporal causal relationship between DDE and caries. Further investigations using longitudinal design are needed to confirm these findings.

## Conclusions

This study has several limitations that need to be taken into account for an adequate interpretation of the results, acknowledging the age of the subjects. Adolescents were recruited from the public high schools only. Only those who signed consent for participation were included to the study, which may cause selection bias. The examinations regarding dental caries should be separated from DDE to avoid observational bias, however, this was not possible in the present study. Some of the subjects would have had their original DDE obliterated by caries, restoration or extraction. Due to the difficulty to differentiate between molars and incisors hypomineralisation and caries, misclassification bias could be taken into account. On the other hand, the tooth- and surface-related recording of MIH-related defects and restorations—which has been used in caries epidemiological trials for decades—is another step forward helping to determine precisely the extent and severity of MIH^37^. It should be emphasized that there is a standardization of the classification used in the study^42,43^. In addition, another limitation of the study is the lack of analysis on access to oral health services, oral health related habits, data on sources of fluoride exposure other than water fluoridation. These factors may be considered as potential effect modifiers that may lead to a weak relationship between dental caries and enamel defects. Also, the reasons for missing teeth were not recorded; hence, some teeth that were missing due to caries were designated as non-carious.

Diagnosis was based on visual and tactile examinations under artificial lighting conditions only. Radiographs were not taken, thus small carious lesions might have been underestimated or not recorded, which is a common problem in cohort studies. Some studies have investigated teeth under natural lighting, others with a flashlight for illumination and have used sterile gauze to remove debris. It is important to mention that in the present study all permanent teeth have been evaluated, while most studies assessed only index teeth. In addition, DMFT was used instead of a more detailed index like ICDAS II. Different indices and criteria, examination variability, methods of recording, and varying age groups in various DDE studies, may have limited the comparisons of the results. Thus, a standardized index should be used in future studies.

Finally, as studies have shown a link between nutrition and DDE and DMFT, a common risk approach should be more rational because nutritional status was not assessed in this study^44^.

We conclude that the results of this study indicated relationship between enamel defects and severity of caries. The presence of qualitative (opacity) DDE has an impact on the severity of caries in 18-year-old adolescents. Finally, it can be concluded that a significant relationship existed between DDE and dental caries in 18-year-old adolescents, with presence of DDE associated with increased caries experience. Nonetheless, further studies are required to explore the relationship in this study population.

## Data Availability

The datasets generated for this study can be released on request to the corresponding author following approval of the Ministry of Health.

## References

[1] Seow WK (2014). Developmental defects of enamel and dentine: Challenges for basic science research and clinical management. Aust. Dent. J..

[2] Anthonappa RP, King NM, Drummond BK, Kilpatrick N (2015). Enamel defects in the permanent dentition: prevalence and etiology. Planning and Care for Children and Adolescents with Dental Enamel Defects Etiology, Research and Contemporary Management.

[3] Casanova-Rosado AJ (2011). Association between developmental enamel defects in the primary and permanent dentitions. Eur. J. Paediatr. Dent..

[4] Olczak-Kowalczyk D (2018). Developmental defects of enamel in the population of Polish adolescents aged 18 years old: The prevalence and selected socio-demographic factors. A cross-sectional study. Nowa Stomatol..

[5] Kühnisch J (2014). GINI-10 Plus Study Group, LISA-10Plus Study Group Genome-wide association study (GWAS) for molar-incisor hypomineralization (MIH). Clin. Oral Investig..

[6] Olczak-Kowalczyk, D. *et al*. Monitoring of oral health. Monitoring of oral health status Polish population in 2016–2020. Assessment of oral health state and its conditions in Polish population at age of 3, 18 and 35–44 in 2017. Medical University of Warsaw, Poland. ISBN-978–83–7637–448–2 (2018).

[7] Pitts NB (2017). Dental caries. Nat. Rev. Dis. Primers..

[8] Vargas-Ferreira F (2015). Association between developmental defects of enamel and dental caries: A systematic review and meta-analysis. J. Dent..

[9] Vargas-Ferreira F (2014). Association between developmental defects of enamel and dental caries in schoolchildren. J. Dent..

[10] Ekanayake L, van der Hoek W (2003). Prevalence and distribution of enamel defects and dental caries in a region with different concentrations of fluoride in drinking water in Sri Lanka. Int. Dent. J..

[11] Machiulskiene V (2009). Prevalence and extent of dental caries, dental fluorosis, and developmental enamel defects in Lithuanian teenage populations with different fluoride exposures. Eur. J. Oral. Sci..

[12] Nota A, Palumbo L, Pantaleo G, Gherlone EF, Tecco S (2020). Developmental Enamel Defects (DDE) and their association with oral health, preventive procedures, and children’s psychosocial attitudes towards home oral hygiene: A cross-sectional study. Int. J. Environ. Res. Public Health..

[13] Fotedar S, Sogi GM, Sharma KR (2014). Enamel hypoplasia and its correlation with dental caries in 12 and 15 years old school children in Shimla, India. J. Indian Assoc. Public Health Dent..

[14] Idicula JJ (2011). Enamel hypoplasia and its correlation with dental caries in school children of Bagalkot, Karnataka. J. Oral Health Community Dent..

[15] Daneshkazemi AR, Davari A (2005). Assessment of DMFT and enamel hypoplasia among junior high school children in Iran. J. Contemp. Dent. Pract..

[16] Seow WK (2011). Comparison of enamel defects in the primary and permanent dentitions of children from a low-fluoride District in Australia. Pediatr. Dent..

[17] Dini EL, Holt RD, Bedi R (2000). Prevalence of caries and developmental defects of enamel in 9–10 year old children living in areas in Brazil with differing water fluoride histories. Br. Dent. J..

[18] Kühnisch J (2018). Relationship between caries experience and demarcated hypomineralised lesions (including MIH) in the permanent dentition of 15-year-olds. Clin. Oral Investig..

[19] Batina N (2004). Ultrastructure of dental enamel afflicted with hypoplasia: An atomic force microscopic study. Calcif. Tissue Int..

[20] Sujak SL, Abdul Kadir R, Dom TNM (2004). Esthetic perception and psychosocial impact of developmental enamel defects among Malaysian adolescents. J. Oral Sci..

[21] Vargas-Ferreira F, Ardenghi TM (2011). Developmental enamel defects and their impact on child oral health-related quality of life. Braz. Oral Res..

[22] Jälevik B, Klingberg G (2002). Dental treatment, dental fear and behaviour management problems in children with severe enamel hypomineralization of their permanent first molars. Int. J. Paediatr. Dent..

[23] Olczak-Kowalczyk, D. *et al*. Monitoring of oral health. Monitoring of oral health status Polish population in 2016–2020. Assessment of oral health state and its conditions in Polish population at age of 6, 10 and 15 in 2018. Medical University of Warsaw, Poland, ISBN: 978–83–7637–484–0 (2017).

[24] Jälevik B, Szigyarto-Matei A, Robertson A (2019). Difficulties in identifying developmental defects of the enamel: A BITA study. Eur. Arch. Paediatr. Dent..

[25] Vandenbroucke JP (2014). STROBE initiative. Strengthening the reporting of observational studies in epidemiology (STROBE): Explanation and elaboration. Int. J. Surg..

[26] WHO: Oral Health Surveys. Basic Methods. 5th ed. (Geneva 2013).

[27] Fédération Dentaire Internationale Commision on Oral Health (1992). Research and epidemiology, a review of the developmental defects of enamel index (DDE Index). Int. Dent. J..

[28] Weerheijm KL, Jälevik B, Alaluusua S (2001). Molar-incisor hypomineralisation. Caries Res..

[29] Lygidakis NA (2010). Best clinical practice guidance for clinicians dealing with children presenting with molar-incisor-hypomineralisation (MIH): An EAPD policy document. Eur. Arch. Paediatr. Dent..

[30] Espelid I, Haubek D, Jälevik B, Koch G, Poulsen S, Espelid I, Haubek D (2017). Developmental defects of the dental hard tissues and their treatment. Pediatric Dentistry: A Clinical Approach.

[31] Steffen R, Krämer N, Bekes K (2017). The Wȕrzburg MIH concept: The MIH treatment need index (MIH TNI). Eur. Arch. Paediatr. Dent..

[32] Ghanim A, Elfrink M, Weerheijm K, Mariño R, Manton D (2015). A practical method for use in epidemiological studies on enamel hypomineralisation. Eur. Arch. Paediatr. Dent..

[33] Szatko F (2004). Oral health of Polish three-year-olds and mothers' oral health-related knowledge. Community Dent. Health..

[34] Olczak-Kowalczyk, D. *et al.* Monitoring of oral health. Monitoring of oral health status Polish population in 2016–2020. Assessment of oral health state and its conditions in Polish population at age of 5, 7 and 12 in 2016 Medical University of Warsaw, Poland, ISBN: 978–83–7637–416–1 (2017).

[35] Americano GC, Jacobsen PE, Soviero VM, Haubek D (2017). A systematic review on the association between molar incisor hypomineralization and dental caries. Int. J. Paediatr. Dent..

[36] Jälevik B, Szigyarto-Matei A, Robertson A (2018). The prevalence of developmental defects of enamel, a prospective cohort study of adolescents in Western Sweden: A Barn I TAnadvarden (BITA, children in dental care) study. Eur. Arch. Paediatr. Dent..

[37] Burns J, Hollands K (2015). Association between developmental defects of enamel and dental caries. Evid. Based Dent..

[38] Nelson S (2010). Dental caries and enamel defects in very low birth weight adolescents. Caries Res..

[39] Wong HM, Wen YF, King NM, McGrath CP (2016). Longitudinal changes in developmental defects of enamel. Community Dent. Oral Epidemiol..

[40] Marshman Z, Rodd HD, Drummond B, Kilpatrick N (2015). The psychosocial impacts of developmental enamel defects in children and young people. Planning and Care for Children and Adolescents with Dental Enamel Defects.

[41] Marshman Z, Gibson B, Robinson PG (2009). The impact of developmental defects of enamel on young people in the UK. Community Dent. Oral Epidemiol..

[42] Lopes LB, Machado V, Mascarenhas P, Mendes JJ, Botelho J (2021). The prevalence of molar-incisor hypomineralization: A systematic review and meta-analysis. Sci. Rep..

[43] Negre-Barber A, Montiel-Company JM, Catalá-Pizarro M, Almerich-Silla JM (2018). Degree of severity of molar incisor hypomineralization and its relation to dental caries. Sci. Rep..

[44] Sheiham A, Watt RG (2000). The common risk factor approach: A rational basis for promoting oral health. Community Dent. Oral Epidemiol..

